# Deformation of Poly-l-lactid acid (PLLA) under Uniaxial Tension and Plane-Strain Compression

**DOI:** 10.3390/polym13244432

**Published:** 2021-12-17

**Authors:** Alina Vozniak, Zbigniew Bartczak

**Affiliations:** Centre of Molecular and Macromolecular Studies, Polish Academy of Sciences, Sienkiewicza 112, 90-363 Łódź, Poland; awozniak@cbmm.lodz.pl

**Keywords:** semicrystalline polymer, deformation mechanisms, PLLA, tension, compression

## Abstract

The ability of PLLA, either amorphous or semicrystalline, to plastic deformation to large strain was investigated in a wide temperature range (T_d_ = 70–140 °C). Active deformation mechanisms have been identified and compared for two different deformation modes—uniaxial drawing and plane-strain compression. The initially amorphous PLLA was capable of significant deformation in both tension and plane-strain compression. In contrast, the samples of crystallized PLLA were found brittle in tensile, whereas they proved to be ductile and capable of high-strain deformation when deformed in plane-strain compression. The main deformation mechanism identified in amorphous PLLA was the orientation of chains due to plastic flow, followed by strain-induced crystallization occurring at the true strain above e = 0.5. The oriented chains in amorphous phase were then transformed into oriented mesophase and/or oriented crystals. An upper temperature limit for mesophase formation was found below T_d_ = 90 °C. The amount of mesophase formed in this process did not exceed 5 wt.%. An additional mesophase fraction was generated at high strains from crystals damaged by severe deformation. After the formation of the crystalline phase, further deformation followed the mechanisms characteristic for the semicrystalline polymer. Interlamellar slip supported by crystallographic chain slip has been identified as the major deformation mechanism in semicrystalline PLLA. It was found that the contribution of crystallographic slip increased notably with the increase in the deformation temperature. The most probable active crystallographic slip systems were (010)[001], (100)[001] or (110)[001] slip systems operating along the chain direction. At high temperatures (T_d_ = 115–140 °C), the α→β crystal transformation was additionally observed, leading to the formation of a small fraction of β crystals.

## 1. Introduction

Poly(l-lactic acid) (PLLA), also called polylactide, has received a lot of attention in recent years due to growing concerns about environmental issues. This polymer can be synthesized from monomers obtained from annually renewable natural resources, e.g., corn, and is biodegradable under industrial composting conditions [1]. Due to its numerous advantages, it turned out to be one of the most promising (bio)polymers that quickly gained an opportunity to become a competitive commodity material.

PLLA is a semicrystalline polymer that can exist in different crystal forms, depending on the conditions of crystallization and/or deformation. The most frequently observed is the stable α phase with a 10_3_ helix, which can exist in two variants: a well-ordered α phase obtained at high crystallization temperature and a less ordered α′ phase (called also δ) formed at temperatures lower than 120 °C [1,2]. Wang et al. [3] argued that the δ-form (α′) is a crystalline phase independent of the α form, not a simple disordered modification of the α form. Two other crystal modifications are based on the 3_1_ helical conformation: a frustrated trigonal β-phase, obtained primarily by drawing amorphous or crystalline (in stable α phase) PLLA at high temperatures and high extensions [4,5,6,7,8], and a γ-phase, to date obtained only by epitaxial crystallization [2]. Moreover, mesophase was also observed in PLLA [2,9]. One of the major disadvantages of PLLA is perhaps its brittle behavior at room temperature. Due to low crystallization rate, an amorphous PLLA is obtained under usual processing conditions. It is brittle and exhibits reduced strength and dimensional stability at room temperature, therefore often requiring modification before application. One of the possible routes of modification is the use of plastic deformation, which may result in modification of the structure and morphology of the polymer, its crystallinity, and the orientation of the crystalline and amorphous phases. This provides an opportunity to significantly improve the physical properties (including mechanical, barrier, and optical) through appropriate mechanical and/or thermal treatment. The mode of deformation selected for such modification and the deformation conditions (such as temperature, deformation rate, and applied strain) strongly affect the features of the obtained material and its final properties. For example, hot- or cold-drawing can be used to orient objects with small cross-sections, thus enabling the production of fibers, tapes, or films. On the other hand, compression, rolling, solid-state extrusion, or die-drawing can be applied to induce chain orientation in objects with large cross-sections, and thus to obtain materials with improved stiffness and strength, suitable for the production of technical parts or other items with larger dimensions, such as, e.g., biocompatible surgical implants. From this point of view, the structural changes and mechanical behavior of PLLA in various deformation modes and under different conditions have recently attracted considerable attention [10,11,12,13,14,15,16,17,18]. However, in spite of quite a large number of papers, still relatively little is known about the mechanical behavior of PLLA in relation to the crystalline structure, its plastic deformation habits, and the mechanisms involved.

The deformation behavior of PLLA was studied mostly in tensile deformation modes—uniaxial [4,9,10,12,19,20,21,22,23,24,25,26,27,28,29,30,31,32,33,34,35,36,37] and biaxial drawing [13,14,38,39,40,41,42], although other deformation modes, such as zone-drawing [43], die-drawing [44], solid-state extrusion [5,6,7,16,17] or plane-strain compression [18] were also explored. It was found that the deformation conditions, such as temperature of deformation, deformation rate, or final strain, seriously affect the structural evolution of PLLA and the resulting mechanical properties; for example, when deformation is performed at a temperature above T_g_, but below the cold crystallization temperature T_cc_ (in this temperature range, the crystallization rate is too low to generate noticeable crystallinity in the time scale of the deformation experiment), the initially amorphous PLLA sample remains amorphous at low and moderate strains and then undergoes strain-induced crystallization when the strain increases [31,40] due to a decrease in conformational entropy associated with molecular orientation that grows gradually with increasing strain. At higher temperatures, above T_cc_, the amorphous PLLA typically crystallizes first as temperature stabilizes prior to deformation, so that the semicrystalline rather than amorphous material is deformed. It implies different mechanisms of deformation employed in both cases, consequently different are the final structure of the material and its properties. Moreover, different crystalline modifications can be formed upon deformation—Hoogsteen et al. [4] found that the α crystal form is obtained by drawing at low drawing rate and temperature, while the β crystals are formed in drawing when a high temperature and drawing rate are employed [4,45]. Later, Stoclet et al. clarified that only the less ordered crystalline α′ form, not α, can be induced upon stretching when T_d_ < 120 °C [27]. On the other hand, Billimoria et al. [37] demonstrated that α crystals can form during drawing at T_d_ ≥ 90 °C. Moreover, at low temperatures close to T_g_, drawing of PLLA frequently leads to formation of mesophase with molecular order lower than in crystals [9,24,29,30]. The strain-induced mesophase was reported to form only when the deformation temperature was not higher than 70–80 °C [9,24]. At higher deformation temperatures (e.g., 90 °C), only crystals of the α′ modification are formed, while the stable α form was not observed at these conditions [9,24,25,27]. Crystals of α–form appear only at higher temperature, T_d_ > 120 °C, probably as a result of the recrystallization process, α′→α. Billimoria et al. [37] suggested, however, that already at T_d_ ≥ 90 °C the ordered crystals of α modification can form directly. In samples initially containing crystallites of only α modification, partial destruction of α crystals and subsequent reorganization into mesophase and/or α′ crystals, depending on the temperature of deformation, was observed at high strains [25]. Such behavior was assumed to imply a strain-induced melting or decrystallization process followed by partial recrystallization at the temperature of the drawing.

The crystals of β phase were first obtained in fibers of amorphous PLLA or those containing the stable α phase, drawn at high temperatures and a high draw ratio [45]. The strain-induced crystal transformation from phase α to β was observed to occur upon solid-state extrusion [5,6,7] and tensile drawing [8], more efficiently at higher temperatures, larger draw ratios, and/or higher draw rate. Sawai et al. [7] demonstrated that α form crystals gradually transform into β-form crystals, and the relative amount of the β-form crystals increases with increasing drawing temperature from 80 °C to 140 °C, reaching a maximum (approx. 0.8) at 130 °C.

Still relatively little is known about the active deformation mechanisms, especially of the crystalline part of PLLA. Generally, crystal rotations and slip were proposed as active deformation mechanisms [22,23]. Another deformation mechanism suggested was the lamellar-to-fibrillar transformation, initiated by crystallographic twinning modes, with little, if any, contribution of crystallographic slip [25]. Moreover, Stoclet et al. [9,24,25] suggested partial destruction of initial crystals of the α′ or α phase and subsequent reorganization into mesophase and/or α′ crystals, depending on the temperature of deformation, i.e., strain-induced ‘melting’ followed by partial recrystallization. Another crystal transformation, α→β, was also assumed without specifying its mechanism (melting-recrystallization or solid–solid transition) [5,6,8]. Moreover, it was suggested that the β-phase is possibly an intermediate phase that could convert into more stable α′ modification [46]. This would explain most of the unusual features of α′ crystallization (as, e.g., increased growth rate, thicker lamellae, and some structural disorder) [2,46]. On the other hand, an opposite transition was postulated by Wasanasuk et al. [47] and by Wang et al. [3]: the strain-induced structural transition from the α to the β form occurring via the transient disordering of the α form to the α′ form (α→α′→β), by assuming the cooperative displacements of the upward and downward helical chains as well as the conformational change.

The limited knowledge about plastic deformation of PLLA demonstrates that a detailed investigation of its deformation behavior is still needed. Therefore, the aim of the present work was to follow the structural changes that occur during the high-strain plastic deformation of PLLA (amorphous or semicrystalline) at various temperatures and to discuss the active deformation mechanisms, including possible differences that can be observed when using different modes of deformation. To avoid unwanted side-effects, plane-strain compression was selected as the primary deformation mode in this study. In addition, samples deformed by uniaxial drawing were also investigated. Plane-strain compression is kinematically similar to tension, both modes leading to an axial plastic flow of the polymer. That flow occurs in the plane-strain compression in the direction perpendicular to the compressive load. However, in contrast to tension, the deformation in this mode is cavity-free and proceeds uniformly and homogeneously over the entire strain range, with no instability such as, e.g., necking, cavitation, or crazing, often observed in drawing. These phenomena could lead to premature fracture of the material. In plane-strain compression the deformation process is not influenced and/or obscured by such side phenomena, and higher strains can be often reached.

## 2. Materials and Methods

### 2.1. Material and Sample Preparation

Polylactide (PLLA) used in this study was commercially available Ingeo^TM^ 7032 D grade, a product of NatureWorks LLC (Minnetonka, MN, USA) containing 97.5% of l-lactide configuration and 2.5% of d-lactide configuration (supplier data). Its density is *ρ* = 1.24 g/cm^3^, the melt flow rate MFR = 15.4 g/10 min (210 °C, 2.16 kg), molecular weight M_w_ = 95,000, and polydispersity M_w_/M_n_ = 1.33 as determined by SEC with a multi-angle laser light scattering (MALLS) detector (Wyatt Technology Corp., Santa Barbara, CA, USA), in methylene chloride.

Prior to processing, the polymer pellets were dried at 100 °C for 4 h under reduced pressure. Then, the rectangular plates of 80 × 80 mm^2^ and 2 or 4 mm thick were produced from dried pellets by injection molding (IM) using a Battenfeld H45210 injection molding machine (Wittmann Battenfeld, Wien, Austria) at 180–210 °C and the mold temperature of 40 °C. The injection rate was 10 cm^3^/s. As revealed by DSC and X-ray diffraction (WAXS, SAXS) the plates obtained by IM demonstrated amorphous structure and nearly no trace of orientation. To obtain semicrystalline samples, the IM plates were cold crystallized by annealing at 122 °C for 1 h. This temperature of annealing was chosen in order to crystallize PLLA entirely in the stable α form, which is known to occur at temperatures above 120 °C [1,2,48]. Further in this article, the amorphous samples are denoted with the symbol ‘A’, while semicrystalline (cold crystallized)—with ‘CC’.

Specimens of a suitable size for the compression experiments in the channel-die were carefully machined from A and CC plates. The cutter and the milled material were cooled with liquid coolant during machining to prevent any unwanted modification to structure of the sample. The specimens had final dimensions of 50 × 25 × 4 mm^3^ along the transverse direction (t.d.), injection direction (i.d.), and normal direction (n.d.) in the injection-molded plates, respectively. The specimens for tensile deformation experiments were cut out of 2 mm thick plates in the form of strips with dimensions 80 × 15 × 2 mm^3^ or in the shape of a dog-bone with a length of the narrow section of 50 mm and its width of 50 mm.

### 2.2. Mechanical Testing

**Figure 1:**
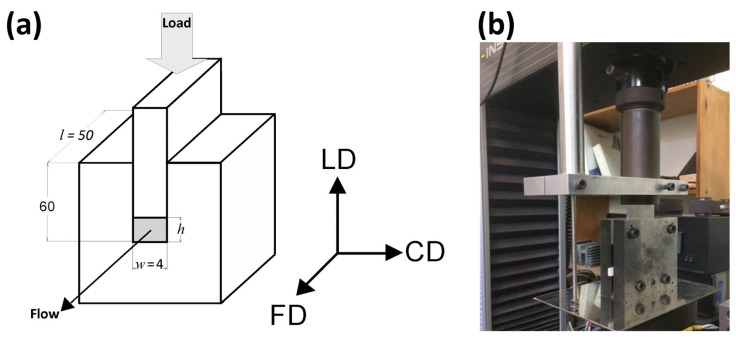
Schematic drawing (**a**) and a photograph (**b**) of the channel-die compression fixture.

Plane-strain compression tests of amorphous (A) and semicrystalline (CC) samples were performed using the loading frame of a universal tensile testing machine (Model 5582, Instron, Norwood, MA, USA) and a compression fixture of the type of channel-die (channel length *l* = 50 mm, width *w* = 4 mm, and depth *h*′ = 60 mm) made of hardened steel and equipped with linear variable differential transformer (LVDT) displacement sensor, mounted close to the specimen for precise strain determination, and two heating blocks with electrical heaters and temperature sensors connected to the temperature controller. Temperature of the die was controlled with the accuracy better than 1 °C. The die, shown schematically in Figure 1, allowed compression of samples up to 50 mm high. In this study, the specimens 25 mm high were used (*l* × *h* × *w* = 50 × 25 × 4 mm^3^). The specimens were precisely machined to exactly match the width and the length of the channel. Specimen surfaces contacting the walls of the die and the plunger were lubricated to reduce friction. Other details are given in [49].

All compression experiments were performed at temperature in the range 70 °C < T_d_ < 140 °C. After the sample was mounted in the channel-die and the setup was heated to the required temperature, it was allowed to equilibrate for an additional 15 min before starting the deformation experiment. All compression tests were carried out with the same constant true strain rate of ė = 0.001 s^−1^, which was controlled by the Bluehill^®^ II software of the testing machine—the current speed of the crosshead was continuously adjusted to the actual height of the specimen in order to keep the true strain rate constant. The true (Hencky) strain was calculated from the reduction in the specimen height (i.e., the size along the loading direction) using the following equation:

(1) $$e=-\int_{h_{1}=h_{0}}^{h_{1}=h}\frac{dh_{1}}{h_{1}}=\ln\left(\frac{h_{0}}{h_{0}-\Delta h}\right)=\ln\lambda$$

where *h*_0_ denotes the initial height of the specimen, Δ*h* is the displacement of the plunger measured with LVDT sensor and *h* = *h*_0_ − Δ*h* is actual specimen height, while *λ* = *CR* = *h*_0_/*h* is the compression ratio. As the sample area under load in a channel-die remains constant (equal to the cross-section of the sample or the plunger) the true stress can be calculated with the following simple formula:

*σ* = *F*/*A* (2)

where *F* is the measured force and *A = l·w* is the surface area of the plunger.

The same universal tensile testing machine, fitted with tension grips and an environmental chamber (Instron, Model 3119) was used for tensile tests, carried out in the temperature range of 70 °C < T_d_ < 140 °C. Tensile specimens, either A or CC, had the form of strips 80 × 15 × 2 mm^3^, with the gauge length of GL = 50 mm (set by the grip distance). The dog-bone shaped specimens were additionally tested when the strips failed in a brittle manner (CC samples). Each specimen was mounted in the grips of the machine once the environmental chamber had warmed up sufficiently, then was allowed to heat up to the desired temperature after the chamber door was closed, and equilibrated for an additional 15 min soaking time before drawing began. Unfortunately, unlike plane-strain compression, the specimens did not deform uniformly when stretched—usually a neck developed during drawing. For this reason, it was not possible to control the strain rate in tension in a manner that keeps the true strain rate constant. Therefore, all tensile tests were performed simply at a constant nominal strain rate of 0.001 s^−1^ (constant crosshead speed 2.5 mm/min). Nominal stress and nominal strain values were used to construct the stress–strain curve. The maximum true strain was calculated from the distance of the ink marks, *l*, printed on the sample surface before deformation, e = ln(*l*/*l_0_*).

The coordinate system used throughout this paper is related to the geometry of the channel-die: the loading direction (LD) is the direction of the compressive force applied to the plunger, the constraint direction (CD) is perpendicular to the sidewalls of the channel, and the flow direction (FD) is parallel to the channel length, i.e., directed towards its opening, as shown in Figure 1. For tensile mode of deformation, the drawing direction is also the direction of the plastic flow, thus it is equivalent to the flow direction in plane-strain compression. Therefore, it was also denoted with the FD abbreviation. The directions perpendicular to FD are the normal direction (ND; normal to the specimen plane), and the transverse direction (TD).

### 2.3. Characterization

#### 2.3.1. DSC

Thermal analysis of PE samples was conducted using differential scanning calorimeter (DSC 2920 (TA Instruments, New Castle, DE, USA)). The melting thermograms were recorded during heating from 0 to 200 °C with the constant rate of 10 °C/min, under a nitrogen flow. The degree of crystallinity *X_c_* was calculated with the equation:

(3) $$X_{c}=\frac{\Delta h_{m}-\Delta h_{cc}}{\Delta h_{m}^{0}}\times 100\%$$

where Δ*h_m_* is the enthalpy of melting, Δ*h_cc_* is the enthalpy of cold crystallization of the sample, both determined from the DSC heating curve, and $\Delta h_{m}^{0}$ = 93 J/g is the enthalpy of melting of 100% crystalline PLLA in the α form [50]. This is the value most often used in the literature, and therefore allows easy comparisons between various reports. However, more recent studies report higher values for this enthalpy, e.g., Righetti et al. [51] estimated $\Delta h_{m}^{0}$ = 143 J/g for the α form, while 107 J/g for the α′ form. Usually, two or three specimens taken from the same sample were tested and evaluated to check the repeatability of the results. The uncertainty in determining crystallinity can be estimated to be less than 1wt.%.

#### 2.3.2. SAXS

The lamellar structure of samples was probed with 2-dimensional small-angle X-ray scattering (2D-SAXS). The 1.2 m long pinhole camera was coupled to a low divergence X-ray CuK_α_ micro source, operating at 50 kV and 1 mA (sealed-tube micro-source integrated with multilayer collimation optics, producing a highly collimated beam with a divergence of 0.8 × 0.8 mrad^2^; GeniX Cu-LD by Xenocs, Grenoble, France). The collimation optics was combined with 2 assemblies of crossed hybrid scatterless slits (Xenocs), placed between the multilayer optics and the sample stage, distanced 1.2 m each from the other. The collimation optics and the slit assemblies formed the well-collimated beam of the square cross-section less than 1 mm^2^. The scattering produced by the sample was recorded with the Pilatus 100 K solid-state area detector of the resolution of 172 × 172 µm^2^ and module size of 83.8 × 33.5 mm^2^ (Dectris, Baden-Daetwill, Switzerland). The long period (LP) was determined from one-dimensional sections (background and Lorentz corrected) of 2D patterns using the Bragg’s law. Other details are given in [52].

#### 2.3.3. WAXS

The distribution of crystallite orientation was evaluated with 2-dimensional wide-angle X-ray scattering (2D-WAXS) in the transmission mode using a flat camera connected to a sealed-tube source, operating at 50 kV and 40 mA (CuK_α_ radiation, *λ* = 0.154 nm; Malvern Panalytical, Almelo, The Netherlands). The 2D-WAXS patterns were collected with a Pilatus 100 K area detector (Dectris, Baden-Daetwill, Switzerland).

**Figure 2:**
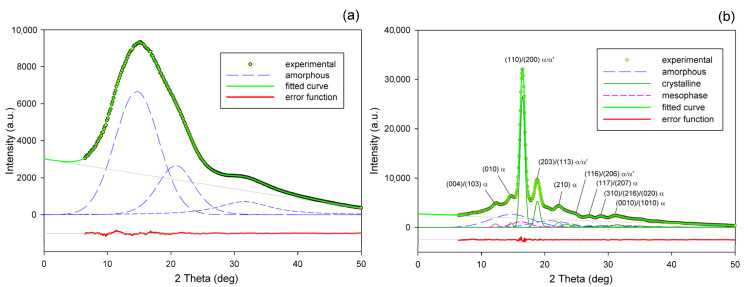
Examples of fitting the amorphous halos and mesomorphic and crystalline peaks to the scattering curve of A (**a**) and CC samples (**b**).

Radial intensity profiles, I(2Θ), were obtained by azimuth integration of the 2D patterns over defined range of azimuth by means of the XRD2DScan, v.4.1.1. software (usually approx. +/−5° from the direction of interest, as FD or CD; 180° to find the average intensity curve). The peak fitting procedure was then applied to these diffractograms for accurate determination of parameters of the crystalline peaks and amorphous halos needed for further estimations, e.g., calculation of crystallinity. The FITYK computer program, dedicated to diffraction peak-fitting [53,54], was used for this purpose. Crystalline diffraction peaks and amorphous halo were fitted with Pearson VII and Gaussian function, respectively. Using the Pearson VII profile instead of Gaussian led to more reliable fits, especially when the presence of mesophase in the sample had to be considered. The weight fraction of identified phases was calculated from the ratio of the contribution of the scattering specific for the respective phase to the total scattering area, summed from the two images that were obtained in the CD- and LD-view. On this basis, the crystallinity of the material was estimated. Figure 2 shows two examples of fitting of azimuth-integrated scattering profiles of amorphous and semicrystalline samples, respectively. The fitting procedure reveals three halos related to amorphous phase, located at approximately 2θ = 14.7–15.0°, 2θ = 20.8–21.2°, and 2θ = 31.0–31.4°, with roughly equal FWHM ≈ 8° (cf. Figure 2a). Similar positions of these halos were reported in the literature [24,34] The merging of the first two halos results in a broad scattering as in PET. According to Stoclet et al. [24] these halos are related to two characteristic interchain spacings in amorphous PLLA, while the third, well separated halo around 2θ = 31°, is related to intrachain spacings—the corresponding distance d ≈ 0.29 nm along the chains precisely matches the characteristic distance between the methyl groups in the 3-fold helix [24]. A mesomorphic order characterized by a well-defined X-ray scattering located at 2θ = 16.2° with a relatively narrow FWHM ≈ 3.5°, irrespective of deformation temperature and strain level, was identified in diffractograms of several deformed samples (cf. Figure 2b). The crystalline peaks observed in semicrystalline samples (Figure 2b) match the positions expected for the α and α′ modifications.

## 3. Results and Discussion

### 3.1. Characterization of the Initial Samples

**Figure 3:**
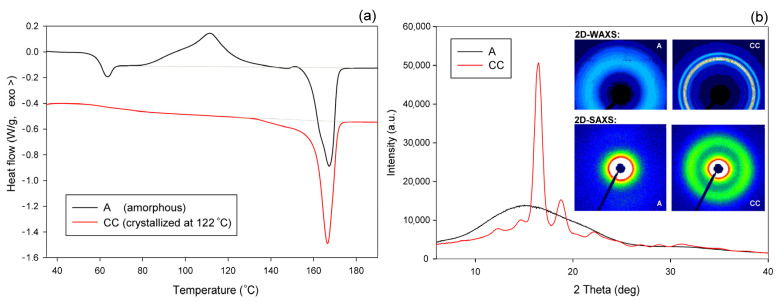
DSC thermograms (**a**) and X-ray diffractograms (**b**) of initial amorphous sample A and semicrystalline sample CC, cold crystallized at 122 °C. Insets show the respective 2D-WAXS and 2D-SAXS patterns.

The A and CC samples used for further experiments (raw amorphous and cold crystallized at *T* = 122 °C, respectively) were characterized by DSC, SAXS, and WAXS. Figure 3a presents the DSC curves, while Figure 3b shows the respective X-ray data—the diffractograms derived from 2D patterns that are shown in the inset of Figure 3b (upper row). The inset also shows, in the bottom row, the 2D-SAXS patterns of samples A and CC. The DSC curve of sample A features clear glass transition followed by a sharp endotherm and two distinct exo- and endothermic peaks related to cold crystallization and melting during specimen heating, respectively. The sharp endotherm just above the glass transition is probably associated with the aging-induced enthalpy relaxation of amorphous polymer. The big endothermic peak actually consists of two overlapping peaks, which are due to recrystallization (α′→α) and the melting of crystals of the α form, respectively [55]. In contrast, thermogram of the CC sample shows only a single melting peak, which indicates the presence of only α modification, expected for samples crystallized above 120 °C [2,48]. The exoterm related to cold crystallization is not observed here, as the sample already crystallized fully during 1 h long annealing at *T* = 122 °C. The change in heat flow related to the glass transition in the CC sample is less pronounced than in the amorphous material due to its high crystallinity. 2D-WAXS images and diffractograms obtained from these patterns through azimuth integration, presented in Figure 3b, reveal the fully amorphous structure of sample A and partially crystalline of cold crystallized sample CC. The amorphous halo, as well as crystal diffraction rings seen in 2D images, appear as uniform rings without changing the intensity along the azimuth. This indicates that both the amorphous and the crystalline phases show no traces of the preferred orientation in either material A or CC. Additionally, 2D-SAXS patterns presented in Figure 2b, showing diffuse scattering in sample A and a uniform ring indicating lamellar ordering in sample CC, do not reveal any orientation of the structure. Several structure-related parameters determined from DSC and X-ray data of Figure 3 are presented in Table 1. It can be seen that sample CC demonstrates glass transition temperature T_g_ slightly higher than amorphous sample A, and an identical maximum temperature of the melting peak, which confirms the α form as the main crystal modification in the CC material. Estimations of crystallinity by both DSC and WAXS methods confirm the practically amorphous structure of sample A (within the scope of the experimental error) and semicrystalline structure of CC with high crystallinity exceeding 50 wt.%.

### 3.2. Deformation Behavior

**Figure 4:**
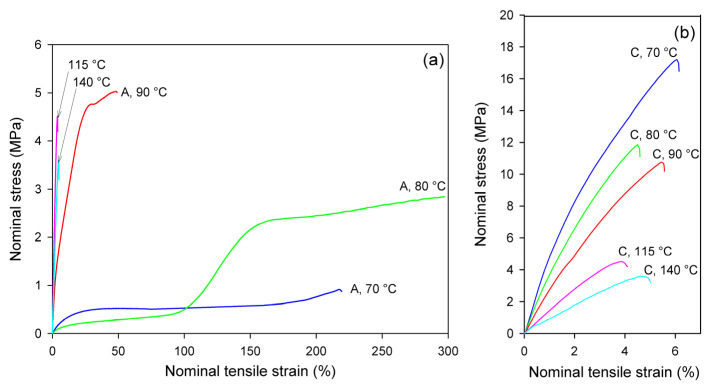
Nominal stress–nominal strain curves obtained in uniaxial tensile tests at temperatures indicated. (**a**) amorphous samples, A; (**b**) cold crystallized samples, CC. Note the different scale on the stress and strain axes in (**a**,**b**).

Figure 4 presents the nominal stress–nominal strain curves of A and CC samples deformed in the uniaxial tension mode at temperatures in the range T_d_ = 70–140 °C. The mechanical response of the initially amorphous A samples is shown in Figure 4a, while the stress–strain relationships of crystalline samples CC are presented in Figure 4b. Initially amorphous A samples subjected to drawing at T_d_ = 70 °C deformed practically uniformly to high strains, while those drawn at T_d_ = 80 °C and 90 °C deformed in a non-uniform manner, developing a distinct neck. The samples deformed at T_d_ = 115 °C and 140 °C demonstrated quasi-brittle behavior and fractured early, before reaching the yield point (cf. Figure 4a). Similar brittle behavior was observed in the semicrystalline CC samples, regardless of the deformation temperature (Figure 4b). As shown in Figure 4a, the nominal strain in ductile samples ranged from less than 50% (T_d_ = 90 °C) to well above 200% (T_d_ = 70 °C or 80 °C). However, due to non-uniform deformation and formation of the neck, the local strain in the region of the greatest deformation within the necked zone was actually much higher; the local strain calculated from the displacement of the ink markers printed on the specimen was approximately ε = 230% at T_d_ = 70 °C, 475% at 80 °C, and 270% at 90 °C. This corresponds to the maximum true strain of e = 1.2, 1.75, and 1.3, respectively.

Glass transition temperature of the amorphous material was detected around 61 °C (see Table 1). In the drawing temperature range 70 °C ≤ T_d_ ≤ 80 °C, i.e., just above T_g_, the rate of the isothermal cold crystallization was so low that the annealing alone, prior to and during deformation, did not lead to noticeable crystallinity and only strain-induced crystals could be generated in A samples in the time scale of the deformation experiment (see DSC results, discussed later). This effect can be clearly observed in samples deformed at 80 °C, where deformation begins in the amorphous material with relatively low stress and the stress jump is observed above ε = 100%, which is related to a significant increase in crystallinity due to strain-induced crystallization. For A samples deformed at T_d_ = 90 °C, the 15 min period of annealing to equilibrate the temperature prior to deformation was long enough to induce cold crystallization, so that deformation began in the already partially crystallized sample. At even higher temperature of deformation (115 °C, 140 °C), the cold crystallization was even faster, and it practically completed before drawing started; therefore, the deformation behavior of A samples at these temperatures was quite similar to that of the fully crystallized CC samples (cf. Figure 4b).

**Figure 5:**
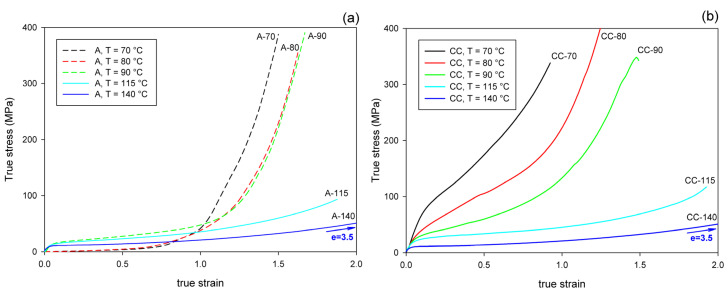
True stress–true strain curves of samples deformed by plane-strain compression in a channel-die at temperatures indicated. (**a**) Amorphous samples, A; (**b**) cold crystallized samples, CC.

Figure 5 presents the mechanical behavior of the studied materials subjected to the plane-strain compression in a channel-die. As both A and CC samples deformed uniformly in the entire strain range, the true stress–true strain curves were much easier to determine than in in the case of tension, and are presented here to illustrate the mechanical response to plane-strain compression.

Importantly, under all tested conditions, the ultimate strain of the compressed samples is much greater than that observed in uniaxial tension, especially when comparing the crystalline samples, which can deform under compression to the true strain of e = 1–3.5, while in tension they demonstrated quasi-brittle behavior and broke at a few percent strain (e < 0.1).

It can be observed that all curves presented in Figure 5 show the same features, typical for plane-strain compression of a polymeric material: the initial elastic range is followed by the region of plastic flow at a moderately increasing stress and then by the range of strain hardening, where the stress rises to very high values, often well above 300 MPa. Generally, the strain-hardening is shifted to higher strain and the ultimate stress tends to decrease with increasing temperature of deformation, in both A and CC samples. In the crystalline CC samples, the level of the stress is the highest at the low deformation temperature of 70 °C and gradually decreases with increasing temperature of deformation. This is mainly due to the plastic resistance of the crystals, which decreases with increasing temperature [56,57].

There is a clear difference in the deformation behavior of A and CC samples when compressed at a relatively low temperature of T_d_ = 70–90 °C. As with the stretched counterparts, the deformation of A sample begins in an almost amorphous material in a rubbery state. Therefore, it responds initially with low stress, until the increasing molecular orientation combined with the effect of temperature brings the onset of strain-induced crystallization, which apparently begins at true strains above e = 0.5. Subsequently, this new crystalline phase, increasing in quantity and already well oriented, causes a significant increase in the stress response of the material, which soon leads to a strong strain-hardening effect. At a higher deformation temperature (T_d_ ≥ 115 °C), the crystalline phase may appear earlier, mainly due to thermal conditions, but it is not so well oriented as compared to that produced by the strain-induced crystallization at lower temperature. This results in a later and weaker strain-hardening effect. In contrast, the crystalline phase in the CC samples is already fully formed, albeit randomly oriented, before any deformation begins. Crystalline lamellae form a quasi-continuous stiff skeleton for the amorphous component. Such a phase structure demonstrates a relatively high resistance to plastic deformation from an early stage, determined by the plastic resistance of the crystals. This leads to high stress at yield and plastic flow. As the deformation progresses, the gradual orientation of the crystallites in the direction of flow due to crystal plasticity causes an increase in stress and ultimately brings the effect of strain-hardening. However, this hardening is not as rapid as in the case of amorphous samples deformed slightly above T_g_, at T_d_ = 70 °C or 80 °C, where a strong strain-induced crystallization was observed.

### 3.3. Thermal Properties

**Figure 6:**
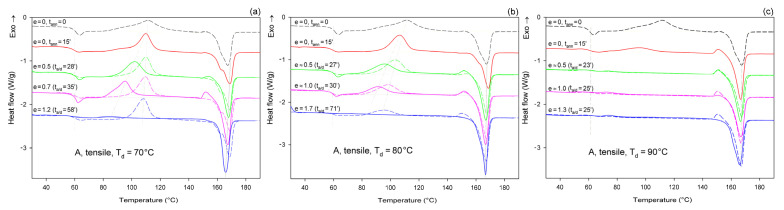
DSC heating scans of initially amorphous samples (A) drawn uniaxially at T_d_ = 70 °C (**a**); 80 °C (**b**); and 90 °C (**c**), to the indicated true strain (solid lines). For each sample before deformation, a soak time of 15 min was used to equilibrate the temperature. The dashed lines represent samples that were annealed only at the respective temperature and time but were not deformed. The total residence time at the deformation or annealing temperature, t_a/d_, is indicated for each curve.

Figure 6 shows the DSC melting thermograms of initially amorphous A samples deformed in uniaxial tension at T_d_ = 70, 80, and 90 °C, respectively. For comparison, thermograms of the initial sample and samples that were only annealed at the same temperature and time as the deformed samples, but actually were not deformed, were plotted with dashed lines.

As can be seen in Figure 6a, the samples that were annealed at T_a_ = 70 °C (dashed lines) show a cold crystallization peak at about 110 °C and a double melting peak with a major maximum around 168 °C, regardless of the annealing time. Such bimodal melting was frequently observed in PLLA when T_c_ was between 100 °C and 120 °C [48,55]. Both endothermic peaks were found to be related to the melting of the α phase: the lower temperature peak is related to the synchronous melting of the original α form crystals and the phase transition from α′ to α, while the higher temperature peak is connected to the melting of the crystals formed during the α′→α crystalline phase transition [48]. The crystallinity of the samples after annealing at T_a_ = 70 °C, calculated from the enthalpy difference related to the melting and cold crystallization peaks, respectively, ranged from *X_c_* ≈ 0.4 wt.% up to approx. 2.3 wt.%. On the other hand, X-ray examination of these samples did not reveal any trace of crystalline phase (*X_c_* = 0%). All these very low values of crystallinity are close to this estimated for the initial A sample before further treatment: *X_c_* ≈ 2 wt.% (see Table 1). This indicates that annealing at 70 °C, i.e., slightly above the T_g_, does not affect the structure of the sample over the time scale of the experiment, and that the sample remains essentially amorphous. On the other hand, samples deformed at this temperature by uniaxial drawing demonstrate a shift of the cold crystallization peak to a lower temperature with an increasing strain and a significant decrease in size of this peak, which finally disappears at the true strain of e = 1.2 (which corresponds to the residence time at the deformation temperature t_a_ = 58 min). The crystallinity of the deformed samples estimated from the enthalpy difference was initially stable at a fairly low level—less than 2 wt.% at e = 0 (t_a_ = 15 min), 4.5 wt.% at e = 0.5, and 5.1 wt.% at e = 0.7—but it increased significantly to *X_c_* = 42.7 wt.% when the true strain reached e = 1.2. This result illustrates the development of the crystalline phase through the strain-induced crystallization process, which apparently takes place at T = 70 °C only in the high strain range. In addition, the post-Tg endothermic peak, similar to the enthalpy relaxation phenomenon observed for aged polymers, appeared at e = 0.5–0.7. According to Stoclet et al. [9,24], this endotherm in deformed samples can result from “melting” of the mesophase that was possibly formed upon drawing.

In samples annealed at T = 80 °C, the cold crystallization peak shifted to a lower temperature, below 100 °C, and decreased in size, while the double melting peak, previously observed in samples annealed at T_a_ = 70 °C, was replaced with a single peak with extended annealing time. Additionally, in samples annealed longer than 15 min, a small exothermic peak emerged just before the melting peak. Such a feature was usually observed in PLLA when T_c_ was below 100 °C and is probably related to the α′ to α phase transition, immediately followed by the melting of the newly formed α phase. This small exothermic peak just before melting—representing the α′→α transition—was found not to be a melt–recrystallization process, but rather a solid phase transition without the melting of the α′-phase [48]. The crystallinity of the annealed samples increased moderately with the annealing time from less than 2 wt.% at t_a_ = 15 min to nearly 21 wt.% at t_a_ = 40 min (lower crystallinity, *X_c_* = 12.4 wt.%, was estimated from X-ray diffraction data). The crystals formed were in both α′ and α form. On the other hand, when the samples were deformed by drawing at T_d_ = 80 °C, the cold crystallization peak shifted to lower temperatures and decreased even faster than in the samples subjected only to annealing, while the small exothermic peak before the melting peak associated with the α′→α transition could already be seen at e ≥ 0.5. A post-Tg endothermic peak, similar to that observed at T_d_ = 70 °C and possibly related to the mesophase, can be recognized at e = 0.5 and 1.0. The crystallinity estimated for A samples deformed by drawing T_d_ = 80 °C increased from a low initial value of about 2 wt.% to *X_c_* = 12.1 wt.% at e = 0.5, next up to 25.8 wt.% at e = 1.0, and further up to 48.9 wt.% at e = 1.75. This crystallinity is much higher than that resulting from annealing alone, so that a significant fraction of crystals must have arisen due to strain-induced crystallization. Comparing the stress–time relationship, which is similar to the nominal stress–nominal strain curve (see Figure 4a) and the change in crystallinity with time, it can be concluded that the upward stress jump in the stress–strain curve observed around the nominal strain of 100% is directly related to an increasing fraction of the well-oriented strain-induced crystalline phase.

Amorphous samples annealed at T_a_ = 90 °C for t_a_ > 15 min show no peak of cold crystallization after heating in DSC, which indicates that the crystallization was complete at this time. The overall crystallinity of the annealed samples increased from about 0% at t_a_ = 0 to approx. 27 wt.% at t_a_ = 15 min and, further, up to more than 40 wt.% at t_a_ ≥ 20 min (crystallinity estimated from X-ray diffraction data: *X_c_* = 0 at t_a_ = 0, *X_c_* = 19.3 wt.% at t_a_ = 15 min, and 37–40 wt.% at t_a_ = 24–40 min). An exothermic peak just before the melting peak associated with the α′→α phase transition and a single melting peak were present in the thermograms, regardless of the time of annealing, which could be expected for T_c_ = 90 °C [48]. This confirms that the crystalline phase formed upon annealing at T_a_ = 90 °C consisted of a mixture of α′ and α form crystals, similar to the samples annealed at 80 °C. Deformation upon drawing of A samples at T_d_ = 90 °C was non-uniform, with the formation of a distinct neck. Therefore, it was possible to find parts deformed to different strains in the same sample. The cold crystallization peak was observed in the deformed material at a temperature only slightly lower than in the annealed only samples, whereas the small exothermic peak before the melting peak associated with the α′→α transition, seen in the annealed samples and in that deformed to e ≈ 0.5, decreased with strain and eventually disappeared at e = 1.3. At the same time, a low temperature shoulder could be recognized in the melting peak of the material deformed to e = 1.3, which suggests that some recrystallization phenomena might occur at high strain. The crystallinity of the stretched samples (T_d_ = 90 °C) increased from approx. 27 wt.% at e = 0 (t_a_ = 15 min) to *X_c_* = 52.3 wt.% at e = 1.3. This crystallinity is notably higher than that resulting from the annealing alone (up to 40 wt.%, depending on the annealing time), so that a significant fraction of crystals must have arisen due to strain-induced crystallization during drawing.

**Figure 7:**
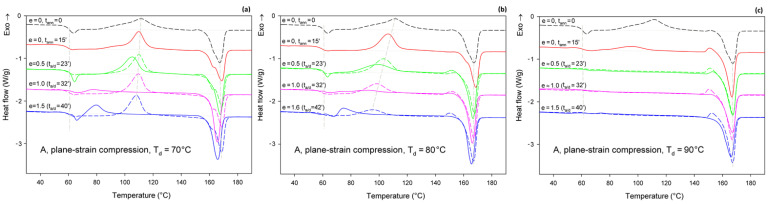
DSC heating scans of initially amorphous samples (A) deformed in plane-strain compression at T_d_ = 70 °C (**a**); 80 °C (**b**); and 90 °C (**c**), to the indicated true strain (solid lines). For each sample before deformation, a soak time of 15 min was used to equilibrate the temperature. The dashed lines represent samples that were annealed only at the respective temperature and time but were not deformed. The total residence time at the deformation or annealing temperature, t_a/d_, is indicated for each curve.

Figure 7 presents the DSC thermograms of initially amorphous A samples, deformed by plane-strain compression in a channel-die at the same temperatures as drawing: T_d_ = 70, 80, and 90 °C. Similar to tension, here thermograms of annealed-only samples are also presented for reference with dashed lines. Thermograms of samples compressed at T_d_ = 70 °C show the same features as those of samples drawn at this temperature (cf. Figure 6a and Figure 7a)—the cold crystallization peak shifts to a lower temperature and decreases in size with increasing strain; a single melting peak (α phase) and a post-Tg endothermic peak (mesophase) can be also seen in the deformed samples. The crystallinity of the compressed samples increases significantly between true strain of e = 0.5 and 1.0—from *X_c_* ≈ 1.4 wt.% to more than 40 wt.%. This indicates an intense strain-induced crystallization in this strain range, which is responsible for heavy strain hardening beginning below the true strain of e = 1 (see Figure 5a).

Additionally, the samples compressed at T_d_ = 80 °C (Figure 7b) and T_d_ = 90 °C (Figure 7c) show thermal behavior similar to the respective tensile samples. The same features can be recognized in both tensile and compressed samples. These include a post-T_g_ endothermic peak, possibly related to the mesophase, which can be observed in samples deformed at T_d_ = 80 °C. This peak, however, does not occur in samples compressed at T_d_ = 90 °C. The crystallinity of the samples compressed at 80 °C, calculated from the difference between the enthalpy of the melting and cold crystallization peaks, increases clearly, from 12.8 wt.% to over 40 wt.% with an increase in the true strain from e = 0.5 to 1.0. This again indicates a significant contribution of the strain-induced crystallization. When compression is carried out at T_d_ = 90 °C, the material shows *X_c_* = 26.9 wt.% before the deformation (after 15 min of soak time) and quickly increases its crystallinity to above 40 wt.% already at e = 0.5 (t_a/d_ = 23 min). This means that deformation proceeded in the semi-crystalline material with a significant crystalline fraction from the very beginning, which explains why the stress observed during the deformation of A samples at low strains is higher at T_d_ = 90 °C than at T_d_ = 70 °C or 80 °C, where the nearly amorphous material is deformed in the initial deformation stages (cf. Figure 4a or Figure 5a).

**Figure 8:**
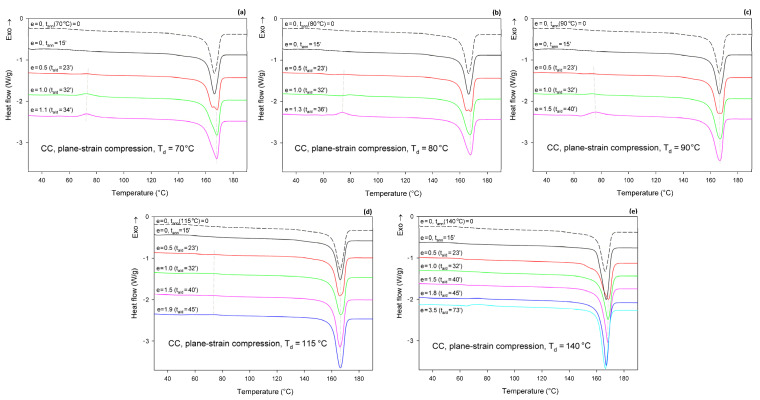
DSC heating scans of semicrystalline CC samples deformed in plane-strain compression at T_d_ = 70 °C (**a**); 80 °C (**b**); 90 °C (**c**); 115 °C (**d**); and 140 °C (**e**) to the true strain indicated. The dashed line shows the thermogram of the initial CC sample, cold crystallized at *T* = 122 °C and not processed further. The total residence time at the deformation temperature, t_a/d_, is indicated for each curve.

Figure 8 shows the DSC thermograms of CC samples (cold crystallized during 1 h annealing at T = 122 °C), compressed in a channel-die at T_d_ = 70, 80, 90, 115, and 140 °C. The thermogram of the initial CC sample is also provided for reference. The thermograms of the initial CC sample, as well as the CC samples subjected to 15 min annealing at any set temperature, show no peak of cold crystallization. A poorly developed glass transition and a single endothermic peak associated with the melting of α form crystals can be observed. These features indicate that annealing at T = 122°C resulted in the highly crystalline material (*X_c_* = 54 wt.%) of pure α form. Thermograms of CC samples that were subjected to plane-strain compression at T_d_ = 70–115 °C show an additional exothermic peak centered well below 80 °C, related to cold crystallization. This peak is generally small; it is barely visible at e = 0.5 and increases only slightly with increasing strain. It was bigger at T_d_ = 70–90 °C than at 115 °C. At T_d_ = 140 °C, this peak was not observed below e = 1.8 (corresponding to the compression ratio of λ = 6). At the same time, the crystallinity calculated from the enthalpy difference decreased with increasing strain from initial 54 wt.% to approx. 47–48 wt.% at the maximum strain when T_d_ = 70 or 80 °C and to about 51 wt.% at T_d_ = 90 °C, or remained roughly constant at T_d_ = 115 °C. In contrast, during deformation at T_d_ = 140 °C, which temperature was notably higher than the annealing temperature of the CC sample, the crystallinity tended to increase with strain (time), which was probably an annealing effect. The observations reported above for T_d_ = 70–115 °C suggest that a limited fraction of the crystalline structure, growing with strain, was destroyed during the deformation process. Interestingly, samples that were deformed to a relative low strain e = 0.5 demonstrated a double melting peak, suggesting that a fraction of the initial α crystals was converted into crystals of the α′ form of relatively high stability, similar to those α′ crystals formed at T_c_ = 100–120 °C together with the crystals of the α form [48]. At higher strains, only a single melting peak can be observed, although this peak demonstrates a remarkable asymmetry, which may suggest a low-temperature shoulder due to the second melting peak, associated with the synchrotronous α′→α phase transition and melting of α crystals.

### 3.4. Structrure and Orientation of the Crystalline Phase (WAXS and SAXS)

**Figure 9:**
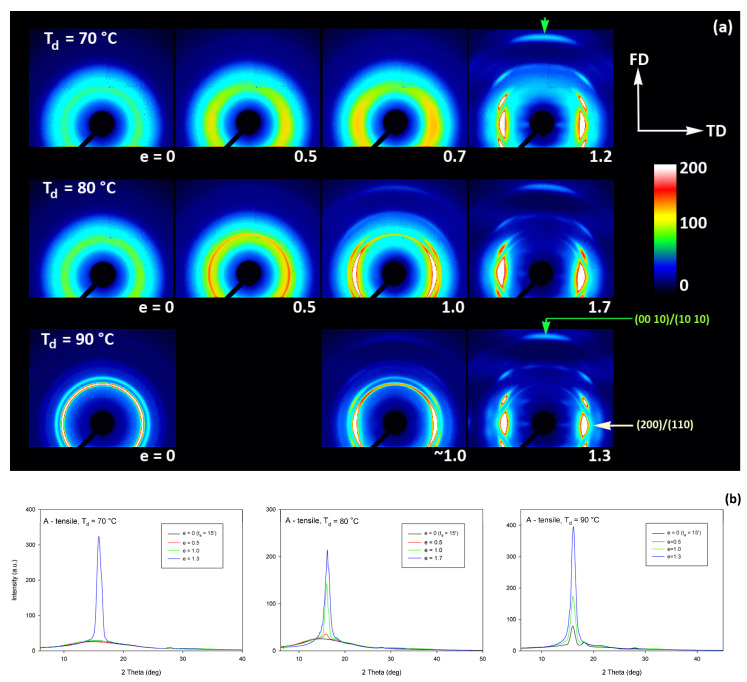
(**a**) 2D-WAXS patterns of the initially amorphous A samples deformed in uniaxial tension at temperature T_d_ = 70 °C (**top row**), T_d_ = 80 °C (**middle row**), T_d_ = 90 °C (**bottom row**) obtained in transmission with the X-ray beam illumination normal to the plane of the sample. The true strain indicated for each pattern is the local true strain calculated from the distance of the ink marks printed on the sample. (**b**) One-dimensional radial sections derived from 2D images along the transverse direction, TD—obtained by azimuth integration of 2D patterns in the azimuth range (−5°, +5°) around TD (ψ_TD_ = 0°).

As already reported in Section 3.2, the initially amorphous samples deformed at temperature T_d_ ≤ 90 °C demonstrated ductile behavior and could be deformed to the true strain exceeding 1.2 (nominal strain ε ≈ 230%). The A samples, when deformed at higher temperatures of T_d_ = 115–140 °C, fractured before reaching the yield point, similarly to CC samples drawn at any temperature. The behavior of A in this temperature range is brittle, similar to CC samples, as the cold crystallization of PLLA is now very fast and almost complete during the soak period before deformation begins. Consequently, the structure of this A sample becomes very similar to that of CC sample, as must their deformation behavior. Figure 9 presents the evolution of the 2D diffraction image with strain observed in the drawn A samples, deformed to the indicated true strain, at temperatures T_d_ = 70, 80, and 90 °C, respectively. It can be noted that all drawn samples described here demonstrated axial symmetry along the drawing direction, FD, as the 2D-WAXS patterns recorded for a given specimen in the front-view (X-ray illumination perpendicular to the sample surface; the patterns shown in Figure 9) and in the edge-view were very similar, almost identical. This symmetry along FD was actually expected as for uniaxial drawing.

The A samples deformed at 70 °C showed initially and at low strains a non-oriented amorphous structure, manifested in the form of a uniform diffuse ring observed in the 2D-WAXS pattern. As the strain increased to e = 0.5 and further to 0.7–1.0 the sample remained essentially amorphous (cf. one-dimensional scans presented Figure 9b) but underwent some molecular orientation along FD, which resulted in a concentration of the scattering along the transverse direction, TD. Finally, at the true strain of e≈1.0, some crystals developed due to strain-induced crystallization. As suggested by the position of the diffraction spots in the 2D-WAXS pattern, these newly formed crystals were already well oriented with their c axis (the chain direction) along the drawing direction. In particular, at 2Θ ≈ 31–32°, in the polar region of the pattern, one can observe the reflections of the (0010) and (1010) planes (2Θ = 30.99° and 32.09°, respectively), which are apparently oriented roughly perpendicular to FD, suggesting a preferred orientation of crystals with the chain direction along FD. Billimoria et al. [37] assigned the peak located above 32° to the (018)_α′_ plane instead of to (1010). However, we observed the peak at approx. 32.1–32.3°, so assigned it to (1010) plane rather than to (018) (expected at 2Θ = 32.09 and 32.80°, respectively). The diffraction observed in the discussed range was assigned in the past to the development of crystals oriented along FD [18] or to the contribution of the amorphous phase strongly oriented along FD—the third amorphous halo at 2Θ ≈ 31° arising from the characteristic interchain spacing of the methyl group in PLLA three-fold helices [24]. However, in the collected data, one can distinguish easily and separate the contribution of amorphous halo from contributions of two crystalline peaks that were assigned here to (0010) and (1010) planes. These peaks can be observed only in these patterns which also show other crystalline reflections, i.e., belong to crystalline samples, while they were not seen in images of oriented amorphous samples, where only the almost uniform ‘intrachain’ amorphous halo was observed. As the normal to the (0010) plane is parallel to the chain direction in the orthorhombic α crystal while the normal to the (1010) plane is close to this direction, a presence and azimuth position of diffraction peaks of assigned to these planes can be used for tracking the orientation of the chain direction in crystals.

Similar orientation behavior was observed in samples drawn at T_d_ = 80 °C and 90 °C, except that the crystalline phase appeared in these samples earlier than during drawing at 70 °C due to cold crystallization associated with annealing, which apparently started earlier than the strain-induced crystallization, occurring only at high strains, as confirmed by the DSC results reported earlier. Similar to the amorphous phase, the orientation of the crystals produced by cold crystallization remains relatively low at strains below e = 1. The preferred orientation of the crystals along FD becomes significantly stronger above e = 1, when the strain-induced crystallization also becomes active.

**Figure 10:**
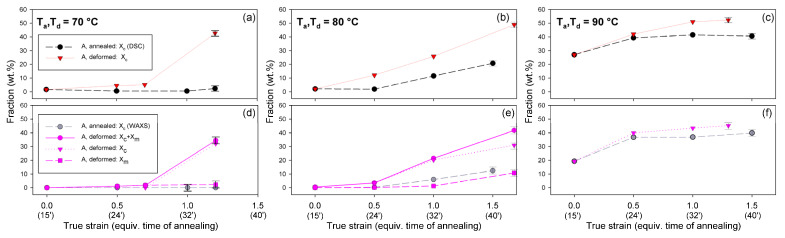
Phase composition (fraction of the crystalline phase and the mesophase) in A samples deformed by uniaxial drawing and the reference samples subjected to annealing at the respective temperature and time equivalent to the time of deformation. (**a**–**c**): composition determined from DSC; (**d**–**f**): composition estimated from X-ray diffractograms through peak fitting procedure. The temperature of deformation or annealing: 70 °C—(**a**,**d**); 80 °C—(**b**,**e**); and 90 °C—(**c**,**f**).

Detailed examination of the diffraction curves extracted from the 2D-WAXS patterns by azimuth integration allowed to determine the phase structure of the deformed samples and to estimate their crystallinity using curve-fitting procedures, as described in the Materials and Methods section. It turned out that, for T_d_ = 70 °C and 80 °C, the ‘best fit’ to the diffraction curves of deformed samples was obtained when the peak located at 2Θ = 16.2° and with a moderate FWHM ≈ 3.5° was taken into consideration, in addition to broad amorphous halos and narrow crystal reflections (cf. Figure 2b). This peak corresponds to the mesophase [9,24,25] developed from the strain-oriented amorphous chains at a strain well above e = 0.5. This result, together with the observation of the post-T_g_ endotherm in thermograms of the respective deformed samples (see Figure 6), which, according to Stoclet et al. [9], may be associated with the “melting” of the mesophase, strongly suggest the formation of the mesophase during deformation at T_d_ = 70 °C or 80 °C. For samples deformed at higher temperature (T_d_ = 90 °C), the fits of diffractograms that did not include the mesophase peak demonstrated comparable or perhaps slightly better quality than those assuming the presence of the mesophase (similar values of the weighted sum of square roots were obtained in both cases). Moreover, the characteristic post-T_g_ endotherm mentioned above was not present in thermograms of the samples deformed at T_d_ = 90 °C (see Figure 6c). Therefore, the formation of the mesophase during drawing at T_d_ = 90 °C cannot be confirmed. This finding is in line with the conclusion of Stoclet et al. [9,24], who observed generation of mesophase at T_d_ = 70 °C and 80 °C and found the upper limit of mesophase stability in PLLA below T = 90 °C. Figure 10 presents the phase composition of the drawn A samples (fraction of crystalline phase and mesophase) determined through the peak-fitting procedure applied to the X-ray diffraction data, compared with the crystallinity estimated from the DSC melting thermograms. A fairly good agreement of the sum of the crystalline and mesophase fraction determined from WAXS and the DSC-based crystallinity can be observed (note that the mesophase present in the sample after its deformation was converted into crystalline phase when heated in DSC). The content of both the crystalline and mesomorphic phases detected in samples deformed at T_d_ = 70–80 °C tends to increase with strain, especially in the range of high strains, above e = 1.0 (corresponding nominal strain, ε = 170%), which confirms that the mesophase as well as crystalline phase were formed from the amorphous phase, presumably well oriented due to plastic flow. Both the meso- and crystalline phase show a preferred orientation along the drawing direction, FD, as indicated by the major reflections concentrated in the equatorial plane of the WAXS pattern. In samples deformed at T_d_ = 70 °C, the mesophase fraction is relatively low, not exceeding 2.5 wt.% with e = 1.2, while at T_d_ = 80 °C this fraction is clearly higher, increasing to over 10 wt.% at e = 1.7. Most likely, the mesophase developed directly from the oriented amorphous phase at the same time as the crystalline component rather than through destruction of the previously formed crystals.

**Figure 11:**
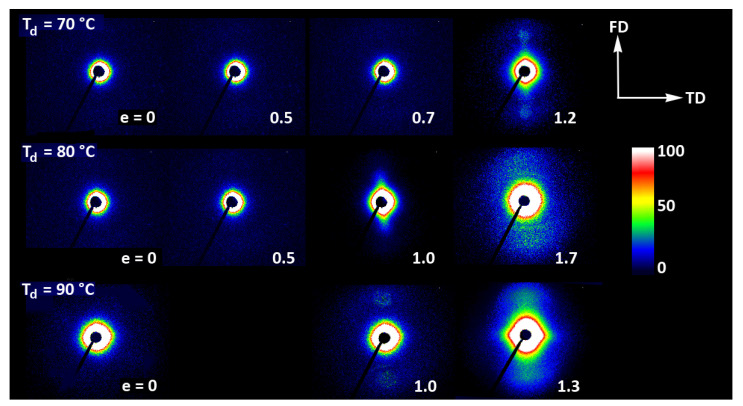
2D-SAXS patterns of the initially amorphous A samples deformed in uniaxial tension at temperature T_d_ = 70 °C (**top row**), T_d_ = 80 °C (**middle row**), and T_d_ = 90 °C (**bottom row**). The true strain indicated for each pattern is the local true strain calculated from the distance of the ink marks printed on the sample.

Figure 11 presents the 2D-SAXS images of the drawn A samples, complementing the 2D-WAXS patterns of Figure 9. It can be seen here that the scattering of the deformed samples that remained essentially amorphous (e.g., e < 1 at T_d_ = 70 °C or 80 °C) is a more-or-less uniform diffuse scattering. As oriented crystals developed in the material due to strain-induced crystallization, two faint peaks emerged in the SAXS patterns along the drawing direction, FD. These maxima indicate some periodic stacking of the lamellar crystals developed along the FD direction. The signal is weak, suggesting that the periodicity of the lamellar structure that was formed during the deformation is rather low. This stacking is perhaps less regular than in cold crystallized CC samples, where, at similar level of crystallinity, stronger scattering due to the lamellar structure was observed.

Strong scattering developed in samples deformed at 80 °C and 90 °C in the final stages of their deformation, at e = 1.3–1.4, in the range of very small scattering angles, which is observed near the beam-stop, especially in the equatorial plane (along TD). This scattering is probably related to cavitation and voiding phenomena preceding fracture or right after sample unloading (samples were probed with SAXS in the unloaded state).

**Figure 12:**
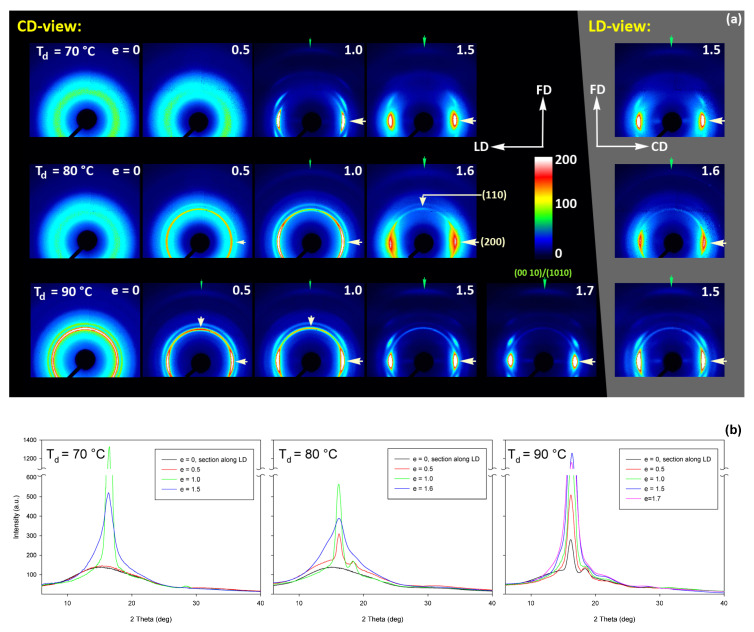
(**a**) Transmission 2D-WAXS patterns of the initially amorphous A samples deformed in the plane-strain compression at temperature T_d_ = 70 °C (**top row**), T_d_ = 80 °C (**middle row**), and T_d_ = 90 °C (**bottom row**), to the indicated true strain. The patterns on the left (on a black background) were obtained in the CD-view (X-ray illumination along the CD, image plane perpendicular to it). The column on the right (gray background) shows the patterns collected in the LD-view. The white arrows indicate positions of the maxima in the (200)/(110) reflection, while the green arrows mark the weak maxima related to the (0010) and (1010) reflections. (**b**) One-dimensional radial sections derived from CD-view images along the loading direction, LD—obtained by azimuth integration of 2D patterns in the azimuth range (−5°, +5°) around LD (ψ_LD_ = 0°).

**Figure 13:**
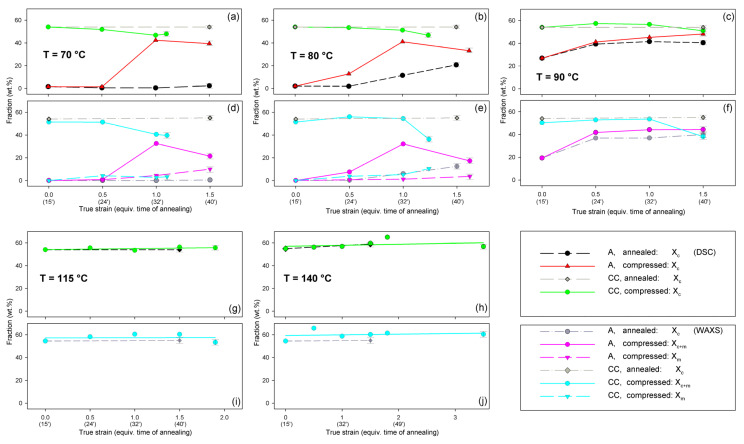
Phase composition (fraction of the crystalline phase and the mesophase) in A and CC samples deformed by plane-strain compression and the reference samples subjected to annealing at the respective temperature and time equivalent to the time of deformation. (**a**–**c**,**g**,**h**): composition determined from DSC; (**d**–**f**,**i**,**j**): composition estimated from X-ray diffractograms through peak fitting procedure. The temperature of deformation or annealing: 70 °C—(**a**,**d**); 80 °C—(**b**,**e**); 90 °C—(**c**,**f**); 115 °C—(**g**,**i**); and 140 °C—(**h**,**j**).

The 2D-WAXS and 2D-SAXS patterns of similar A samples, but now deformed by plane-strain compression, are presented in Figure 12 and Figure 13. Only the samples deformed in the temperature range of 70–90 °C are presented here as, as already discussed, at higher temperatures the cold crystallization of PLLA was so fast that the A sample quickly converted to CC, even before deformation began. Consequently, the deformation behavior of A had to be almost identical to that of CC sample, discussed later in this section.

The WAXS images of Figure 12, recoded for compressed samples, are qualitatively similar to the respective images of the tensile samples shown in Figure 9. Both sets show similar features. Interestingly, the images recorded with X-ray illumination along the constrained direction (CD-view) and those along the loading direction (LD-view) are very similar. This suggests an axial symmetry of the crystalline texture along the flow direction, FD, similar to that observed earlier in tensile samples. Such a uniaxial texture was surprising, as the strong constraints imposed on the sample by the walls of the channel lead to a triaxial stress state under load, therefore development of rather triaxial texture with preferred orientation direction and preferred plane could be expected, as, e.g., the (100)[001] texture (quasi-single crystal) observed in polyethylene [57]. Apparently, when the sample was initially amorphous and isotropic, its deformation resulted primarily in an axial flow that led to molecular orientation along FD and consequently to strain-induced crystallization, fed by well oriented chains, resulting in uniaxial crystalline texture, which was not modified markedly by the stress distribution in transverse directions due to lateral constraints. The resultant crystal orientation is quite distinct. Examination of the strongest reflection, associated with (110) and (200) planes, along the azimuth demonstrated that normals to the (200) plane (2Θ = 16.46°) are oriented preferentially in the equatorial plane (LD-CD), while the normals of the (110) plane (2Θ = 16.32°), which is tilted approx. 60° from (200) in the unit cell, form a broad and much lower maximum centered along FD. Moreover, the reflection of the (0010) and (1010) planes, directly illustrating the orientation of chain direction [001], can be also seen in the FD direction. All these features suggest a relatively sharp uniaxial crystalline texture with the chain direction oriented preferentially along the drawing direction, FD. Such a sharp texture is due to strain-induced crystallization that converted highly oriented amorphous material into crystals oriented along FD, and probably not due to the activity of any crystallographic mechanism that would cause the crystallites to progressively rotate towards FD.

Figure 13 compares the results of determination of the phase structure and crystallinity of the compressed samples, based on DSC and WAXS data. As with the tensile samples, the curve-fitting procedure was applied to the diffractograms. As can be seen, there is a clear agreement between DSC- and WAXS-based estimates. The crystallinity of compressed A samples increases with strain up to e = 1.0, similarly to the respective tensile samples (cf. Figure 10). The same mechanisms are responsible for such structural evolution: the strain-induced crystallization leading to highly oriented crystals, which prevails at T_d_ = 70–80 °C, and the cold crystallization (effect of annealing) only supported by the strain-induced crystallization at T_d_ = 90 °C, resulting in an overall less oriented material. This orientation, however, increases later with increasing strain due to activation of deformation mechanisms characteristic for semicrystalline polymers. At e ≥ 1.0, a decrease in crystallinity was detected by both DSC and WAXS. This is probably a result of a partial destruction of the crystalline ordering due to the fragmentation of the lamellae—see the SAXS results presented later in this section.

Similar to the drawn samples, the ‘best fit’ to the diffraction curves of samples deformed in a channel-die at T_d_ = 70 °C and 80 °C was obtained when mesophase formation during deformation was assumed, which resulted in the appearance of the peak at 2Θ = 16.2° and FWHM ≈ 3.5°. For higher deformation temperatures, the fits not taking into account the mesophase demonstrated comparable (T_d_ = 90 °C) or better quality (T_d_ = 115–140 °C) than those assuming the presence of the mesophase, hence the appearance of the mesophase in this temperature range was unlikely. This finding again confirms the upper limit of mesophase stability in PLLA below *T* = 90 °C, suggested by Stoclet et al. [9]. In A samples deformed at T_d_ = 70 °C or T_d_ = 80 °C the mesophase fraction increases with the increase in strain, although it remains at a low level of a few percent, similar to the tensile samples. Most probably, the mesophase developed during the deformation directly from strongly oriented amorphous chains, simultaneously with the crystalline phase (strain-induced crystallization), which is seen clearly up to the strain e = 1.0. At higher strains, a certain reduction in crystallinity was observed, therefore it is possible that apart from these strongly oriented amorphous chains, the remnants of damaged or destroyed crystals may also participate in the formation of a new mesophase fraction [9,24,25].

**Figure 14:**
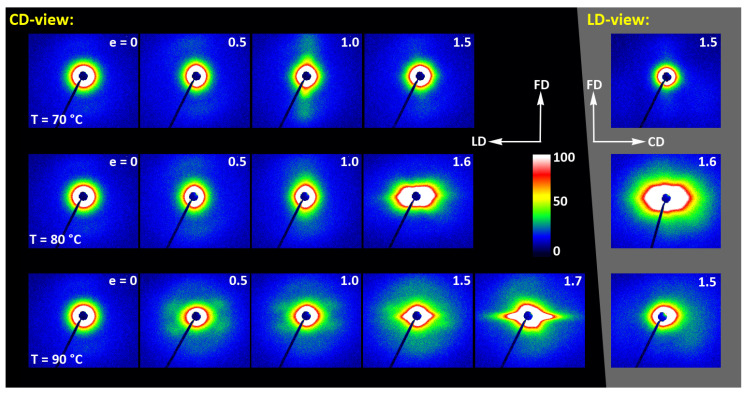
2D-SAXS patterns of the initially amorphous A samples deformed in the plane-strain compression at temperature T_d_ = 70–90 °C (**rows from top to bottom**), to the indicated true strain. The patterns on the left (black background) were obtained in the CD-view. The patterns in the column on the right (gray background) are LD-view.

Figure 14 presents a set of 2D-SAXS images of A samples compressed in a channel-die. It can be noticed that the patterns of samples compressed at T_d_ = 70 °C, when the developed crystallinity is relatively low, are similar to those observed for drawn samples (cf. Figure 11)—for low strain, the pattern reveals mainly the features of amorphous material, although a very weak signal related to lamellar ordering can be distinguished, manifested by two extremely low maxima along the flow direction, FD (difficult to visualize in the image, but visible in its 1D cross-section along FD). The maxima are very low as the crystallinity of the sample is still low, below 2wt.%. At e = 1.0 these maxima become stronger and can already be observed directly in the pattern, while they seem to fade out again when the strain increases to e = 1.5, which is probably related to a partial loss of crystallinity, decreasing from approx. 42 wt.% at e=1.0 to about 39 wt.% at e = 1.5 (DSC estimates). As with the corresponding tensile samples, the low scattering intensity at the maxima discussed here, even at e = 1.0, suggests that, in addition to the low crystallinity, the periodicity of the lamellar structure that was created during deformation due to strain-induced crystallization, must be also rather poor. Comparing the patterns recorded in the CD- and LD-view, one can notice their similarity, which indicates the axial symmetry of the structure, when probed at the level of the lamellar structure. This symmetry is in fact analogous to that described earlier for the lower level of the crystalline texture.

Similar evolution of the lamellar structure was observed in samples compressed at T_d_ = 80 °C. In this case, however, in addition to the features discussed above, the characteristic hexagon-like shaped scattering can be seen in the strongly deformed sample (e = 1.6) at the lowest angles in SAXS images captured in both CD- and LD-view. The strength and angular position of this scattering component suggest that it is related to cavitation and voiding phenomena preceding fracture or following unloading. In such a case, these hypothetical voids should be elongated and oriented at an acute angle with respect to the flow direction in order to produce scattering of the observed shape. These might be voids and fissures located in the interlamellar layers, while lamellae had been tilted due to their shearing or sliding, i.e., the mechanisms known as active in deformation of semicrystalline polymers [57].

At T_d_ = 90 °C, the amorphous material started to crystallize already during the 15 min soaking period prior to deformation, and demonstrated a crystallinity about 27 wt.% at the start of deformation, increasing further up to approx. 40 wt.% so that the deformation took place in a semicrystalline material from the very beginning. At e = 0, a scattering in the form of a weak yet uniform ring indicated that the initial orientation of lamellae was random. When the strain approached e = 0.5, the SAXS image transformed into a weak four-point type pattern, which suggests the development of a preferred orientation of lamellae with their normals oriented preferentially around 60° from the flow direction. A similar four-point pattern was also observed at higher strains, e = 1.0–1.5, however the angle of the preferred orientation with respect to FD gradually increased to over 75°—i.e., the lamellae tilted progressively towards FD and eventually oriented almost along FD. The development of such a preferred orientation was probably due to inter- and intralamellar shear that is commonly observed in other semicrystalline polymers subjected to plastic deformation [57]. Another feature was observed above e = 1.0—two weak scattering spots appearing in the direction of FD. Such a signal is usually a signature of lamellae fragmentation and their restructuring into a new arrangement with the long period along FD [52,58,59]. Finally, the strong scattering component developed at the lowest angles near beam stop, preferably along LD, at e ≥ 1.5. This scattering is probably related to cavitation and voiding preceding fracture or to voids and fissures parallel to LD formed upon sample unloading.

The long period was determined from the one-dimensional cross-sections of the 2D-SAXS images (CD-view) along the flow direction and the load direction, respectively. It turned out that, for all deformation temperatures, the long period along FD remains approximately constant with increasing strain, at the level of 13–14 nm, while the long period along LD tends to decrease slowly with strain from less than 13 nm to approx. 11 nm. This behavior probably reflects a squeezing of amorphous material from the interlamellar layers when oriented roughly normal to LD, due to the compressive loading. This can happen when the crystallinity is low, as in the case of T_d_ = 70 °C or 80 °C, and the separated lamellar stacks are embedded in a continuous amorphous matrix. When the crystallinity was higher and the crystalline lamellae formed a semi-continuous structure, as in the case of T_d_ = 90 °C, such squeezing is unlikely and the observed reduction in LP may be then the result of lamellae thinning that accompanies crystallographic (interlamellar) slip [57].

The deformation behavior of semicrystalline CC samples, obtained by annealing amorphous samples, deformed in the plane-strain compression mode, was studied in a wider temperature range, T_d_ = 70–140 °C, than amorphous A samples, which, at temperatures 115–140 °C, crystallized so fast they converted to CC material even before beginning deformation. In contrast to uniaxial tension, in which all CC samples easily fractured in a rather brittle fashion, regardless of the deformation temperature (cf. Figure 4b), they were able to deform plastically to high true strains ranging from e = 1.1 at T_d_ = 70 °C to above e = 3.5 at T_d_ = 140 °C in the plane-strain compression (Figure 5b).

**Figure 15:**
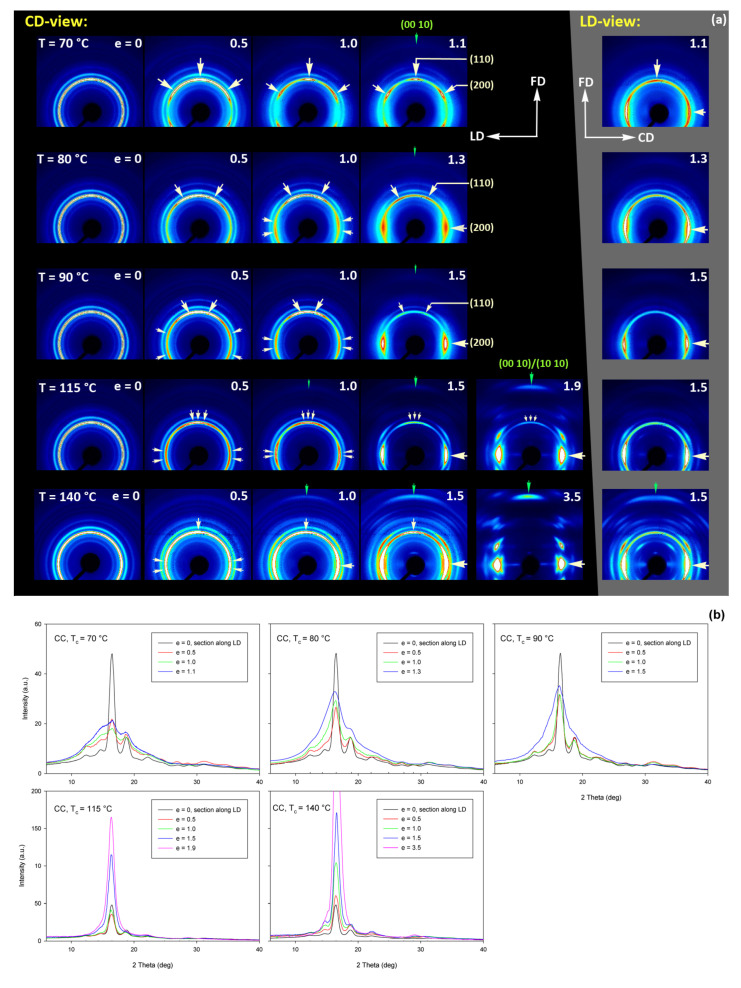
(**a**) Transmission 2D-WAXS patterns of the cold crystallized CC samples deformed in the plane-strain compression at temperature T_d_ = 70–140 °C (**rows from top to bottom**), to the indicated true strain. The patterns on the left (on a black background) were obtained in the CD-view. The column on the right (gray background) shows the patterns collected in the LD-view. The white arrows indicate positions of the maxima in the (200)/(110) reflection, while the green arrows mark the maxima of the (0010)/(1010) reflection. (**b**) One-dimensional radial sections derived from CD-view images along the loading direction, LD, obtained by azimuth integration of 2D patterns in the azimuth range (−5°, +5°) around LD (ψ_LD_ = 0°).

Figure 15 shows the results of WAXS examination of the compressed samples. As the initial crystallinity of the CC samples was high (54 wt.%, as estimated on the basis of DSC results), one can observe development of the preferred orientation of the crystalline phase due to deformation already at the low strains. The evolution of the crystal orientation can be easily observed by following the strain-related changes in the intensity distribution in (200)/(110) reflection along the azimuth in 2D-WAXS images. The (200) and (110) planes in α or α′ modifications of PLLA demonstrate similar interplanar distance, resulting in very close diffraction angles (e.g., 2Θ = 16.32° and 16.46°, for (200)_α_ and (110)_α_, respectively) and consequently in merging their reflections into a single peak. In the oriented material, the maximum of this peaks can slightly shift towards lower or higher angles for a particular orientation of the sample with respect to the X-ray beam, depending on which plane contributes more to the peak. This allows the contribution of the (200) and (110) planes to 2D pattern to be distinguished.

At a low deformation temperature of 70 °C, the (200)/(110) diffraction ring, initially uniform in undeformed sample, developed a maximum along the flow direction in both the LD- and CD-view pattern, already at e = 0.5. Two clearly lower and very broad culminations, centered at approx. 45° from FD, can be also seen in the CD-view pattern. The maximum along FD was identified as related to the (110) plane, while the two tilted contributions were probably associated with the (200) plane. As the strain increased to e = 1.0–1.1, the (110) reflection was still observed along FD, while the (200) maxima turned to the position approx. 58° from FD in the CD-view pattern, sharpened, and increased in intensity to the level of the maximum associated with the (110) plane. The observed tilt angle of 58 °C corresponds well to the angle between normals of (200) and (100) planes in the unit cell (60.3°), thus the maxima in (200)/(110) diffraction observed in the CD-view pattern may originate from the same component of crystal orientation with the (110) plane normal to the FD and a chain direction along CD, i.e., perpendicular to FD. In the LD-view pattern, still relatively broad (200) maxima developed near CD, while a tilted orientation was not observed. Additionally, at e = 1.1, which exhibited the ultimate strain for this deformation temperature, a weak diffraction arc appeared at 2Θ ≈ 32° at the position near FD. This spot, related to reflections of the (0010) and (1010) planes, can be considered as the signature of an additional new component of the crystalline texture, with the chain direction preferentially oriented along FD. The described distribution of crystal orientation developed in CC samples deformed at T_d_ = 70 °C with the (110) planes oriented preferentially perpendicular to FD as the main component of texture, may probably be a result of interlamellar slip (shear), perhaps with some, rather limited, contribution of crystallographic (interlamellar) slip. Stoclet et al. [25] suggested that the twinning mechanism, occurring in (200) or (110) plane in crystals that are oriented with these planes roughly normal to FD, may also be active at the initial stages of deformation at such a relatively low temperature.

At T_d_ = 80 °C, two very broad maxima in (200)/(110) ring, about 25–30° from FD, developed in the CD-view pattern at e = 0.5. Similar, although much weaker maxima can be observed in the LD-view. At e = 1.0, these maxima seen in the CD-view developed further at approx. 25° from FD and could be identified as corresponding to the (110) plane. Meanwhile, the maxima related to the (200) plane emerged near LD, about 80° from FD, and turned further towards LD and increased in intensity as the true strain increased to e = 1.3. It was observed that similar maxima due to diffraction on the (200) planes developed in the LD-view pattern along the CD direction, perpendicular to FD, while the maxima of (110) gradually faded out. As in the case of samples deformed at 70 °C, weak diffraction arcs (albeit notably stronger than those seen at T_d_ = 70 °C) associated with (0010) and (1010) planes appeared in the direction of FD, which may indicate an increasing fraction of crystals oriented preferentially with the chain direction along FD, probably due to growing contribution of the crystallographic slip mechanism to the deformation process. It seems justified, as the critical shear stress for slip in the polymer crystal (plastic resistance) decreases strongly with temperature [56].

A similar evolution of 2D-WAXS patterns was observed at T_d_ = 90 °C. The first maxima in (200)(110) ring appeared now, at e = 0.5, closer to FD and CD, respectively, than at low temperature: stronger maximum, related to the (110) plane, approximately 10° from FD, and weaker, associated with the (200) plane, close to CD—approx. 75–80° away from FD. As the strain increased, these maxima rotated to approx. 68° from FD and to 90° (i.e., towards CD), respectively. While the intensity of the maxima near LD increased, it became significantly weaker near FD, especially at e = 1.5. Meanwhile, in the LD-view patterns, the (110) maxima near FD also decreased notably in intensity and only strong maxima near CD could be eventually observed at e ≥ 1.0. The strong (200) diffraction perpendicular to FD, seen in both CD- and LD-view images, together with the (0010)/(1010) reflection observed along FD, indicates the preferred orientation of crystals with the chain direction along the flow direction, which was probably produced with a noticeable contribution of crystallographic slip mechanisms [57] in addition to interlamellar shear.

At T_d_ = 115 °C and 140 °C, almost the same features could be observed in 2D-WAXS images, although the maxima related to diffraction in the (110) plane seemed weaker, while those associated with the (200) plane became stronger than at lower deformation temperatures, and developed gradually closer into FD and CD, respectively. Moreover, the intensity of the (0010)/(1010) diffraction, observed at e ≥ 1.0, became notably higher, and this diffraction spot could be recognized easily in the patterns as early as at e = 1.0, especially in samples deformed at a high temperature of 140 °C. This indicates an increasing preferred orientation of the crystals with the chain direction along the flow direction, hence the probably increasing contribution of crystallographic slip mechanisms to the deformation process with the increase in the deformation temperature.

Figure 13, presented earlier, reported the changes of phase composition with temperature and strain. As with the samples of series A, in the CC samples, a small amount of mesophase, formed during deformation, was detected when the deformation took place at T_d_ = 70–80 °C, i.e., below the upper limit of mesophase stability. A low fraction of mesophase was detected already at e = 0.5. For the true strain e = 1.0, it was still below 4–5 wt.%, but increased even up to 10 wt.% at higher strains. At the same time, in the deformed samples, a clear reduction in the content of the crystalline phase was observed, which may indicate that, in addition to the mesophase, which was probably formed from oriented amorphous chains, the other fraction, appearing at higher strains, was probably formed from still oriented small residues survived from the crystalline lamellae that were damaged or destroyed during deformation due to deformation instabilities [52,59]. Indeed, Stoclet et al. [9,24,25] suggested a partial destruction of initial α or α′ crystals and subsequent reorganization into mesophase and/or α′crystals, depending on the deformation temperature, i.e., the mechanism of the strain-induced “melting” followed by partial recrystallization. Interestingly, when the deformation temperature was high (T_d_ = 115–140 °C), not only was the mesophase not detected, but no reduction in crystallinity with strain was observed—for a given deformation temperature, the crystallinity estimated on the basis of either WAXS or DSC data remained almost constant over the entire strain range. This may indicate that the crystalline phase did not deteriorate during deformation at these temperatures, or that partial destruction (strain-induced ‘melting’) did indeed occur in this temperature range as well, but that immediate recrystallization took place, keeping the sample crystallinity constant. The SAXS results discussed later in this section suggest that the second scenario, in line with the Stoclet’s hypothesis, is true.

**Figure 16:**
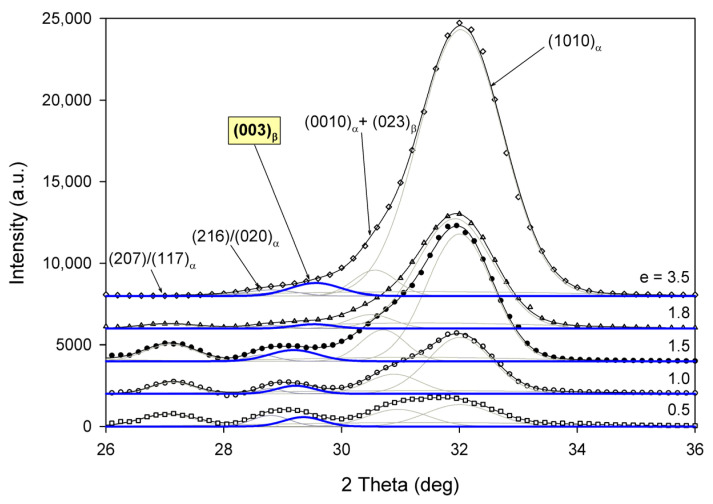
WAXS meridional scans of the 2D-WAXS patterns of samples deformed at T_d_ = 140 °C to indicated strains. The experimental curves (black symbols) are compared with the ‘best fit’ curves (black lines) and their peak components (gray lines). The (003)_β_ peak is drawn with thick blue line.

Another crystal transformation, α→β, was postulated to occur during deformation of PLLA, [5,6,8]. Lotz suggested that the β-phase is possibly an intermediate phase that could convert into more stable α′ modification [46]. On the other hand, Wang et al. [3] explained formation of the β-modification as the strain-induced structural transition from the α to the β form occurring via the transient disordering of the α form to the α′ form (α→α′→β), by assuming the cooperative displacements of the upward and downward helical chains, as well as the conformational change. The crystals of the β-phase were first obtained by drawing fibers at high temperatures and high draw ratios [45]. The crystal transformation from α to β was observed to occur upon tensile drawing [8] and solid-state extrusion [5,6], more efficiently at higher temperatures, larger draw ratios and/or higher draw rates [4]. We examined our data for the α→β transformation and presence of β-phase crystals in deformed samples. As the main (200)_β_ X-ray reflection strongly overlaps with (200)/(110) peak of α modification, the activity of the α→β crystal transformation can be alternatively confirmed by checking the presence of the (003)_β_ reflection from β crystals at 2Θ ≈ 29.8° and, if found, by following the relative intensity of this reflection and the (0010)_α_ or (1010)_α_ reflections from α crystals in the WAXS meridional scan [6,8] (one-dimensional section of the CD-view image along FD), as shown in Figure 16. Careful examination of the experimental data, again employing the peak-fitting procedure, indicated the presence of a very small (003)_β_ peak in the CC samples deformed at T_d_ = 115 °C and 140 °C, which can confirm the small quantities of β crystals formed during deformation at these relatively high deformation temperatures, perhaps by transformation from crystals of the α–phase. At the lower deformation temperature, the β peak was hardly detected, which indicates that α→β transformation was most probably not active then.

Unfortunately, the relative amount of β crystals could not be directly correlated with the ratio of the intensities of (003)_β_ and (0010)_α_ reflections [8]. Nevertheless, the low intensity of (003)_β_ compared to (0010)_β_ or (1010)_α_ suggests a very small amount of β-phase produced, so it can be concluded that the α→β transformation plays only a secondary role in the plastic deformation process. 

**Figure 17:**
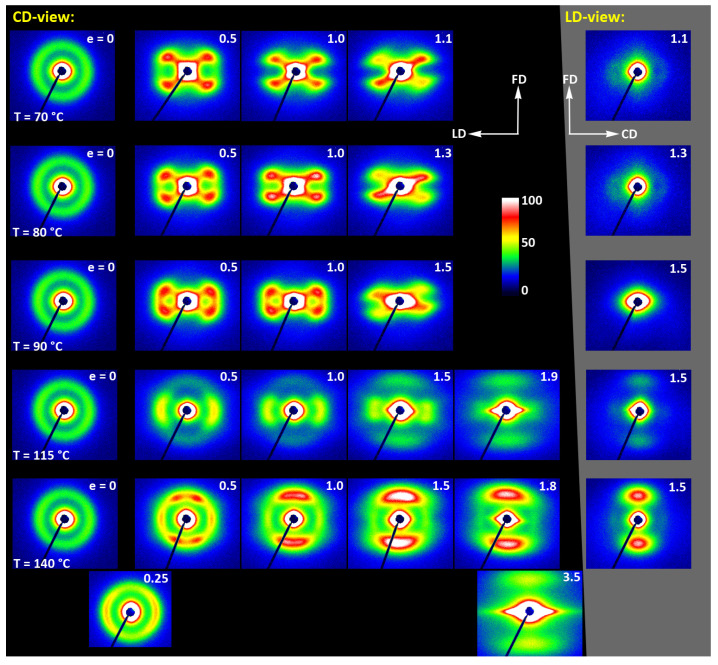
2D-SAXS patterns of the initially amorphous A samples deformed in the plane-strain compression at temperature T_d_ = 70–90 °C (**rows from top to bottom**), to the indicated true strain. The patterns on the left (black background) were obtained in the CD-view. The patterns in the column on the right (gray background) are LD-view.

Figure 17 shows 2D-SAXS images of deformed CC samples. As the crystallinity of these samples is high, they all give a clear SAXS image. In general, the uniform ring observed for the initial non-oriented sample is transformed into a four-point pattern already at the true strain e=0.5, regardless of the deformation temperature. This temperature, however, affects the azimuthal position of the maxima in that pattern—the angle between azimuth at which the maximum is observed and the flow direction changes from ψ ≈ 54° at T_d_ = 70 °C to ψ ≈ 77° at T_d_ = 115 °C. At T_d_ = 140 °C, two broad maxima oriented along LD, i.e., perpendicular to FD, are visible (additionally, another, stronger four-point feature is also observed in this image near FD. This component of the pattern will be discussed later). Most likely, each of these wide maxima is the result of merging of two maxima belonging to the four-point pattern, similar to that observed at lower temperatures, yet now oriented very close to LD (ψ→90°). The formation of this component of the pattern was observed near LD already at e = 0.25, when the second four-point component near FD had not yet appeared. The maxima in the four-point pattern rotated slowly away from FD towards LD when the true strain increased to about 1.5, whereas the intensity at these maxima decreased, especially noticeably faster when e ≥ 1.0. The long period determined along the direction defined by the position of maxima near LD practically did not change with strain when T_d_ = 70–90 °C and it decreased slowly with increasing strain when T_d_ = 115–140 °C. The rotation of the maxima in SAXS pattern away from FD suggests the progressive tilting of lamellae towards the plane containing FD as the strain increased. The long period remaining constant at low deformation temperatures may suggest that such lamellar rotation was primarily due to interlamellar slip, i.e., the shear taking place in amorphous layers between lamellae [57,60], which proved to be the dominant mechanism at low temperatures. Changes in crystal orientation deduced from 2D-WAXS images (Figure 15) support this view, although a limited contribution of crystallographic slip and twinning was also considered. At higher deformation temperature, the long period in the tilted lamellae considered here tends to slightly decrease with strain. Thinning of interlamellar layers due to interlamellar shear is unlikely, therefore for the observed shortening of the long period, the lamellae has to become thinner. A probable deformation mechanism that could lead to the described orientation of the lamellae and their thinning is the crystallographic chain slip [60], which increasingly supported the main deformation mechanism through interlamellar slip. The intensity of the chain slip increases with temperature as the plastic resistance of polymer crystals decrease strongly with increasing temperature [56]. The emerging preferred orientation of the chain direction in the crystals along the FD direction, as shown by the WAXS results, fully supports this hypothesis. As the easy slip planes are generally the closest packing planes, i.e., of large interplanar distance [60], a suggested active crystallographic slip may be (010)[001], (100)[001], or (110)[001] slip system (i.e., a slip proceeding in the (010), (100), or (110) plane along the [100] direction), as those close-packed planes exhibit the largest interplanar distance of 0.62 nm and around 0.54 nm, respectively.

At the deformation temperature of 140 °C, SAXS images for e = 0.5 and 1.0 seem to consist of two superimposed four-point patterns, each perpendicular to the other. That which formed first, oriented similarly to those observed at lower temperatures, was described above. Its intensity decreased with increasing strain and gradually faded away until it finally disappeared at approx. e = 1.5. The second four-point component with strong maxima oriented near FD increased the intensity to e = 1.5. These maxima turned toward FD with increasing strain and eventually merged at about e = 1.5 creating a strong two-point pattern oriented along FD. A similar, even sharper two-point feature could be observed in the image captured in the LD-view. The origin of this second component is not clear. It can be guessed that the lamellae contributing to such a pattern, strongly deviating from FD, acquired this preferred orientation owing the joint action of crystallographic and interlamellar slip, perhaps with a stronger contribution of the crystallographic mechanism, but it occurred in another population of lamellae of different initial orientation than those contributing to the first component of the SAXS pattern.

At strains above 1.5, the maxima observed in the image along FD become significantly weaker in intensity and, moreover, above e = 1.8 they jump to a new position at a larger angle, which indicates the formation of a new long period along FD, yet shorter than the original. Such a transition is usually a signature of heavy fragmentation of lamellae already severely deformed by slip processes and restructuring the small crystalline residues that have left such a fragmentation to a new ordering giving new long period along FD [52,58,59]. These recreated crystals are smaller and possibly stacked less regularly than the original lamellae, thereby reducing the intensity of scattering. Usually, this new structure can be improved by annealing. The fragmentation process requires that lamellae deformation by chain slip is active and well advanced, as fragmentation usually results from deformation instabilities in the lamellae that have already been significantly thinned by an advanced slip action [52,59]. Similar lamella fragmentation probably also occurred at T_d_ = 115 °C and possibly at 90 °C, above e = 1.5, as suggested by the relatively weak scattering streak appearing along FD in both CD- and LD-view images at e ≥ 1.5 (clearly visible at T_d_ = 115 °C).

## 4. Conclusions

The deformation behavior of PLLA (of amorphous or semicrystalline structure) was studied in a wide range of temperatures above the glass temperature, T_d_ = 70–140 °C. The results presented in this paper allowed us to identify the active deformation mechanisms and compare them for two different modes of deformation: uniaxial drawing and plane-strain compression.

It was found that amorphous PLLA can be easily deformed to high strains if the deformation temperature is relatively low (70–80 °C), when the rate of cold crystallization is slow. Both drawing and plane-strain compression then lead to significant orientation of the amorphous chains, which is followed by the strain-induced crystallization—at the true strain above e = 0.5 (elongation ε = 65%), well-oriented chains are transformed to oriented mesophase (up to approx. 5 wt.%) and oriented α′ crystals, which can later be converted into crystals of α phase. This mechanism turned out to be the primary mechanism leading to high molecular orientation in the drawn amorphous samples. An additional fraction of the oriented mesophase may arise in the high strain range of from crystals damaged by severe deformation. It was confirmed that the upper temperature limit for mesophase stability is below 90 °C.

At higher temperatures, T_d_ ≥ 90 °C, cold crystallization becomes fast enough to convert the amorphous material into semicrystalline material just at the beginning of the deformation experiment. As a consequence, the deformation behaviors of initially amorphous and semicrystalline samples become very similar to each other in this temperature range. Contrary to the amorphous PLLA drawn at low temperatures, the semicrystalline samples proved to be incapable of plastic deformation in tension and demonstrated brittle behavior, regardless of the drawing temperature.

In contrast to uniaxial tension, a well-advanced plastic deformation was observed in plane-strain compression over the entire temperature range, both in amorphous and semicrystalline PLLA. Apparently, the compressive stress component prevented any premature failure phenomena, such as, e.g., crazing or cavitation, and allowed undisturbed plastic deformation up to high strains. This demonstrates that when high plastic deformation is needed (e.g., to modify the properties of the product made of PLLA), the compression modes can appear more advantageous compared to stretching.

The deformation mechanism of amorphous samples compressed at a low temperature (T_d_ ≤ 90 °C) was the same as that found in tensile samples—the orientation of amorphous chains and strain-induced crystallization. For semicrystalline PLLA, obtained either by cold crystallization prior to deformation or crystallizing just at the beginning of the deformation experiment (T_d_ > 90 °C), the interlamellar slip (i.e., the shear in amorphous layers separating lamellae) was identified as the major deformation mechanism. It is supported by crystallographic chain slip and perhaps by twinning. The contribution of crystallographic slip in the deformation process increases noticeably with increasing temperature due to decreasing plastic resistance of crystals. The most probable crystallographic slip systems active in the process are (010)[001], (100)[001], or (110)[001] slip systems, all operating along the chain direction [001].

Apart from the mesophase, which is produced at high strains from residues of the damaged or destroyed crystalline phase at T_d_ < 90 °C, the crystal transformation α→β was also observed, but at high deformation temperature T_d_ = 115–140 °C and high strains. This transformation produces only a small fraction of β crystals in plane-strain compression.

## Figures and Tables

**Table 1:** The characteristics of initial amorphous (A) and cold crystallized (CC) samples.

Sample	T_g_ (°C)	T_cc_ (°C)	T_m_ (°C)	Δ*h_c_* (cc) ^(1)^ (J/g)	Δ*h_m_* (m) ^(2)^ (J/g)	*X_c_* (@R.T.) (wt.%)		LP (nm)
						DSC ^(3)^	WAXS ^(4)^	
A	60.7	111.7	167.5	36.5	38.5	~2	0	-
CC	~62.7	-	167.3	0	50.2	54.0	51.6	18.6

^(1)^ The heat of fusion determined from the cold crystallization peak; ^(2)^ The heat of fusion determined from the melting peak; ^(3)^ Crystallinity of the sample estimated for the initial state of the sample, prior to heating in DSC, calculated from the difference of enthalpies related to melting and cold crystallization; ^(4)^ Crystallinity of the initial samples calculated from X-ray diffraction data, *X_c_* = ΣI_cryst_/(ΣI_cryst_ + ΣI_amorph_).

## Data Availability

The data presented in this study are available on request from the corresponding author.
